# Editorial: ImmunoPET imaging in disease diagnosis and therapy assessment

**DOI:** 10.3389/fmed.2023.1231525

**Published:** 2023-06-20

**Authors:** Francisca Mulero

**Affiliations:** Spanish National Cancer Research Center, Madrid, Spain

**Keywords:** ImmunoPET, antibody, PET imaging, radiopharmacy, imaging, diagnosis

The world of medicine is continuously evolving, constantly seeking novel and more effective diagnostic and therapeutic methods. One of the most promising advancements in recent years is the development of ImmunoPET imaging—a powerful tool that harnesses the specificity of antibodies and the sensitivity of positron emission tomography (PET) to visualize and quantify biomarkers *in vivo*. This cutting-edge technique has the potential to revolutionize disease diagnosis, therapy assessment, and patient outcomes. In this ImmunoPET imaging editorial, we will delve into recent research to provide an overview of how ImmunoPET is shaping the future of medicine and explore its applications in various diseases (1, 2).

## Introduction to ImmunoPET imaging

ImmunoPET imaging combines the high specificity and selectivity of monoclonal antibodies with the sensitivity and quantitative capabilities of PET imaging. This innovative approach offers a non-invasive, real-time assessment of disease processes at a molecular level, providing valuable information that can guide diagnosis, monitor disease progression, and evaluate the efficacy of various therapies. ImmunoPET imaging has been extensively studied in the context of oncology, but its potential applications extend to other areas, such as neurology and immunology (2, 3).

## Antibodies and PET imaging: a perfect match

**Figure 1:**
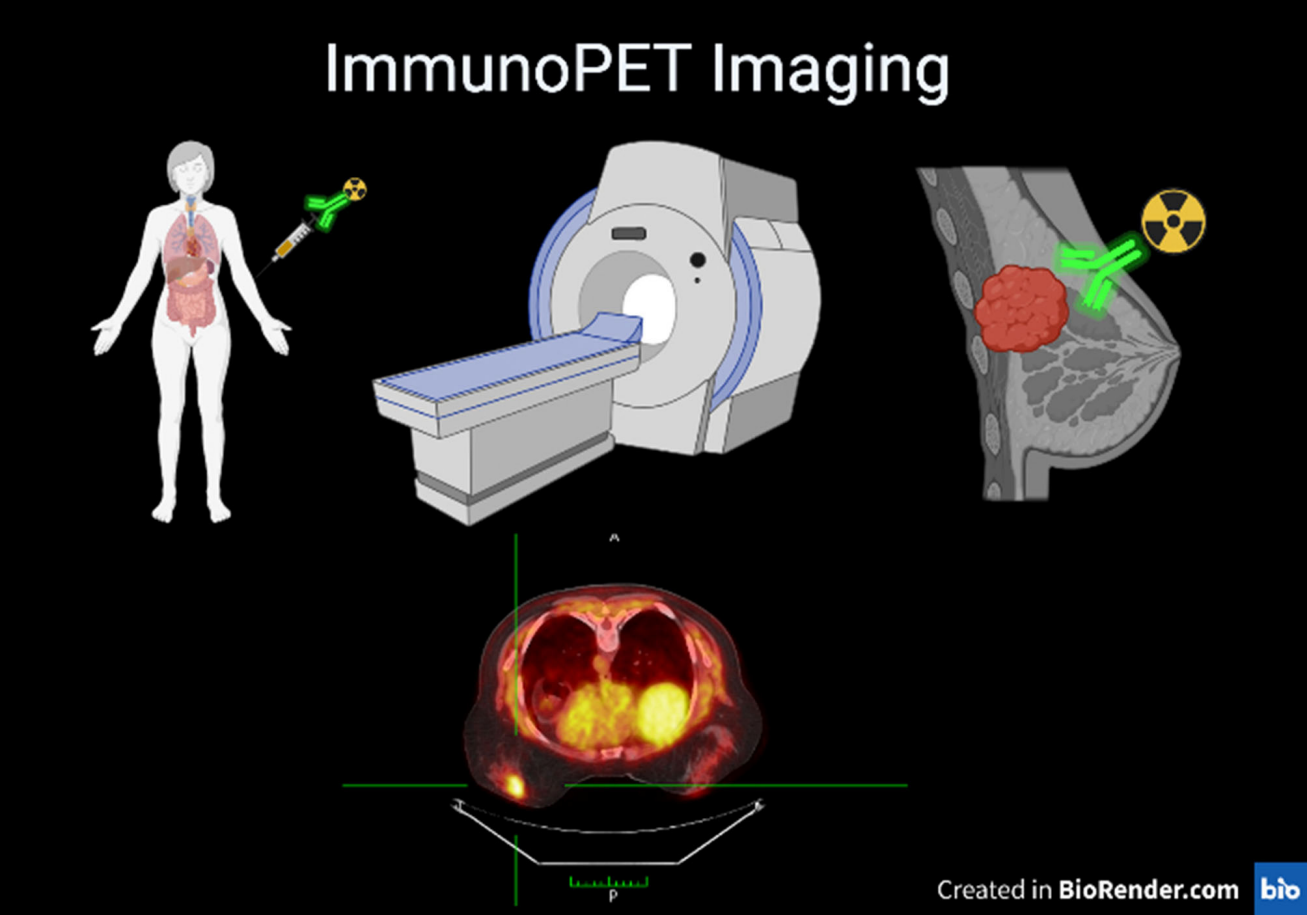
Representative scheme of ImmunoPET technique a patient who is injected with an antibody labeled with a radioactive isotope on the left, a PET device and the breast tumor on the right labeled by the combo Antibody PET isotope. Lower panel PET image showing the breast cancer.

The effectiveness of ImmunoPET imaging lies in its ability to harness the power of both antibodies and PET imaging. Monoclonal antibodies are highly specific and selective for their target antigens, allowing for precise detection of biomarkers implicated in various diseases. On the other hand, PET imaging offers high sensitivity and quantitative capabilities, enabling the visualization and measurement of these biomarkers *in vivo*. By combining these two technologies, ImmunoPET imaging offers a powerful tool for disease diagnosis and therapy assessment (2–4) (Figure 1).

In a recent review published in Frontiers in Medicine as part of the Research Topic *ImmunoPET imaging in disease diagnosis and therapy assessment* (Lugat et al.).

The authors explore the design options and clinical proof-of-concept studies of ImmunoPET tracers. They highlight the importance of optimizing the design of ImmunoPET probes for improved imaging performance and clinical translation. They also discuss the clinical utility of ImmunoPET in various diseases, including cancer, infectious diseases, and autoimmune disorders.

## ImmunoPET imaging applications

One of the most promising areas of ImmunoPET imaging application is in oncology, where it has shown potential in tumor detection, monitoring, and treatment response assessment. Recent studies have explored the use of ImmunoPET imaging in various cancer types, including lung cancer, breast cancer, and hematological malignancies.

As part of same Research Topic published in frontiers in Medicine a group published promising results in the diagnosis of Triple-Negative Breast Cancer (Mulero et al.).

This article explores the use of ImmunoPET imaging in detecting triple-negative breast cancer (TNBC), a particularly aggressive subtype with limited targeted therapies. In this study, researchers developed a nanobody-based PET imaging strategy targeting membrane type 1-matrix metalloproteinase (MT1-MMP), a protein associated with tumor malignancy, progression, and metastasis. Their results demonstrated the precise tumor-targeting capacity of the radiolabeled nanobodies in a TNBC mouse model, highlighting the potential of ImmunoPET imaging as a diagnostic tool based in nanobodies new approach able to access more easily than full standard antibodies.

In the same collection we could find an overview of the Oncological application of ImmunoPET (Manafi-Farid et al.) the authors delve into the application of ImmunoPET in solid tumor imaging. They discuss the challenges associated with antibody-based PET imaging and provide insights into the development of novel imaging agents. This comprehensive review emphasizes the potential of ImmunoPET as a non-invasive imaging modality for precise tumor characterization and treatment response evaluation.

ImmunoPET imaging has also been utilized in the field of immunology to visualize and quantify immune cell populations and their interactions with other cells and tissues. This information can help to elucidate the complex immune processes involved in various diseases and inform the development of targeted immunotherapies.

In recent years, immune checkpoint inhibitors have emerged as a promising class of cancer immunotherapies. ImmunoPET imaging has been employed to visualize and quantify the expression of immune checkpoint molecules, such as PD-L1 and CTLA-4, in tumors and their microenvironment. This information can help to identify patients who may benefit from immune checkpoint inhibitor therapy and monitor their response to treatment.

In a recent study performed in lung cancer and published in the same special collection number (Krache et al.), researchers evaluated the pharmacokinetics, biodistribution, and dosimetry of a murine anti-PD-L1 antibody radiolabeled with zirconium-89 in healthy mice and immunocompetent mice with lung cancer. Their findings demonstrated that tumor uptake occurred within 24 h post-injection, with the best imaging time at 48 h. This study highlights the potential of ImmunoPET imaging in assessing tumor PD-L1 expression in lung cancer, which could improve patient selection for immunotherapy treatments.

The application of CAR T-Cell Therapies in Hematological Malignancies is a very promising therapy approach. Chimeric antigen receptor (CAR) T-cell therapies have emerged as a breakthrough treatment for hematological malignancies, but challenges such as relapse and unique toxicities remain.

This article included in the Research Topic discusses the potential of ImmunoPET imaging to address these challenges by providing a non-invasive platform for longitudinal monitoring of target antigen expression and CAR T-cell pharmacokinetics (Mulgaonkar et al.). By leveraging ImmunoPET imaging, clinicians may be able to identify resistance mechanisms and toxic events early, enabling more effective therapeutic interventions and improved patient outcomes.

## Challenges and future directions

Despite the significant progress made in the field of ImmunoPET imaging, several challenges remain to be addressed. One of the primary limitations is the relatively slow pharmacokinetics and prolonged circulation half-life of full-length antibodies, which can lead to high background signals and reduced image quality. Novel strategies, such as the use of nanobodies or engineered antibody fragments, are being explored to overcome these limitations and further enhance the performance of ImmunoPET imaging.

Moreover, the development of novel targeting agents, such as immune checkpoint inhibitors and CAR T-cell therapies, has opened new avenues for ImmunoPET imaging applications. By combining these innovative treatments with ImmunoPET imaging, researchers and clinicians can obtain a wealth of information on disease processes and treatment response, ultimately leading to more effective and personalized therapeutic strategies for patients.

In this editorial the articles reviewed collectively, demonstrate the growing importance of *ImmunoPET imaging in disease diagnosis and therapy assessment*. They highlight the potential of ImmunoPET as a versatile imaging tool for characterizing diseases at a molecular level, monitoring treatment responses, and guiding personalized therapies. However, challenges such as tracer design, radiochemistry, and clinical translation remain, emphasizing the need for further research and collaboration among scientists, clinicians, and industry partners.

The articles published in this special collection of Frontiers in Medicine contribute significantly to our understanding of ImmunoPET imaging and provide a solid foundation for future advancements in this field. As ImmunoPET continues to evolve, its integration into clinical practice holds great promise for improving patient outcomes, enabling early detection, and facilitating targeted therapies. Continued research and investment in ImmunoPET imaging are crucial to unlock its full potential and bring this technology closer to routine clinical use.

## Conclusion

ImmunoPET imaging represents a groundbreaking approach in disease diagnosis and therapy assessment, offering a non-invasive, real-time assessment of molecular processes *in vivo*. By harnessing the specificity of antibodies and the sensitivity of PET imaging, ImmunoPET has the potential to revolutionize patient care and outcomes in various fields, such as oncology, neurology, and immunology. As researchers continue to explore and refine ImmunoPET imaging techniques, we can expect to see even greater advancements in our understanding of disease processes and the development of targeted therapies in the years to come.

## Author contributions

FM have made significant contributions to the review of the five papers published in Frontiers in Medicine on the topic of ImmunoPET imaging. FM played a pivotal role in conceptualizing the editorial and outlining the structure and scope of the review. Their expertise in molecular imaging and precision medicine provided a solid foundation for the analysis of the ImmunoPET imaging technique and its clinical application.
